# Rice SUV3 is a bidirectional helicase that binds both DNA and RNA

**DOI:** 10.1186/s12870-014-0283-6

**Published:** 2014-10-14

**Authors:** Narendra Tuteja, Mohammed Tarique, Renu Tuteja

**Affiliations:** International Centre for Genetic Engineering and Biotechnology, Aruna Asaf Ali Marg, New Delhi, 110067 India

**Keywords:** ATPase, Mitochondrial protein, *Oryza sativa*, Plant DNA and RNA helicases, SUV3, Unwinding

## Abstract

**Background:**

Helicases play crucial role in almost all the nucleic acid metabolism including replication, repair, recombination, transcription, translation, ribosome biogenesis and splicing and these processes regulate plant growth and development. It is suggested that helicases play essential roles in stabilizing growth in plants under stress because their presence in the stress-induced ORFs has been identified. Moreover in a recent study we have reported that SUV3 helicase from *Oryza sativa* (OsSUV3) functions in salinity stress tolerance in transgenic rice by improving the antioxidant machinery. SUV3 helicase has been identified and characterized from yeast and human systems but the properties and functions of plant SUV3 are poorly understood.

**Results:**

In this study, the purification and extensive characterization of recombinant OsSUV3 protein (67 kDa) is presented. OsSUV3 binds to DNA and RNA and exhibits DNA as well as RNA-dependent ATPase activities. It also contains the characteristic DNA and RNA helicase activity. OsSUV3 can use mainly ATP or dATP as energy source for the unwinding activity and it cannot unwind the blunt-end duplex DNA substrate. It is interesting to note that OsSUV3 unwinds DNA in both the 5’-3’ and 3’-5 directions and thus its activity is bipolar in vitro. The Km values of OsSUV3 are 0.51 nM and 0.95 nM for DNA helicase and RNA helicase, respectively.

**Conclusions:**

This study is the first direct evidence to show the bipolar DNA helicase activity of OsSUV3 protein. The unique properties of OsSUV3 including its dual helicase activity imply that it could be a multifunctional protein involved in biologically significant process of DNA and RNA metabolisms. These results should make significant contribution towards better understanding of SUV3 protein in plants.

**Electronic supplementary material:**

The online version of this article (doi:10.1186/s12870-014-0283-6) contains supplementary material, which is available to authorized users.

## Background

Helicases are highly conserved ubiquitous motor proteins involved in almost all the nucleic acid metabolic processes. They unwind nucleic acid duplexes with affiliated NTP hydrolysis and play essential roles in replication, DNA repair, recombination, transcription, translation, pre-mRNA processing and RNA degradation [1]. Helicases are crucial tools for machinery of the cell. Most helicases contain conserved helicase motifs which are grouped together for the enzymatic and other functions [2,3].

The product of the *SUV3* (suppressor of Var 3) gene was first described in yeast *Saccharomyces cerevisiae* [4]. This gene encodes a DNA/RNA helicase belonging to the Ski2 family of DExH/D-box helicases. The human nuclear *SUV3* gene (*SUPV3L1*) encodes an NTP-dependent RNA/DNA helicase (SUV3p, hSUV3p), which is related to the DexH/D (Ski2p) super family. The gene has been conserved during evolution and is present in purple bacteria, plants, *C. elegans*, Drosophila, mammals and in all eukaryotes [5]. In humans the hSUV3 protein is localized predominantly in the mitochondrial matrix [6]. The human hSUV3 protein is also present in the cell nucleus and was found to have several interacting partners: HBXIP [7], BLM helicase, and WRN helicase [8]. hSUV3p has been reported to unwind both DNA and RNA duplexes and DNA/RNA hybrids and its activity towards DNA is much stronger [6,9]. It was reported that the hSUV3 helicase interacts with replication protein A and flap endonuclease 1 in the nucleus [10]. The yeast SUV3 has been reported to be involved in RNA turnover, mtDNA replication and maintenance of mtDNA stability [11].

To the best of our knowledge there are very few reports on plant SUV3. It was reported that nuclear-encoded *Arabidopsis thaliana* SUV3 (AtSUV3) is localized in *Arabidopsis* mitochondria and possesses ATPase activity [12]. In a recent study we have reported that OsSUV3 dual helicase functions in salinity stress tolerance by maintaining photosynthesis and antioxidant machinery in rice (*Oryza sativa* L. cv. IR64) [13]. Earlier we reported only presence of helicase and ATPase activities in the OsSUV3 protein but the detail characterization has not been reported yet. Here we report extensive characterization of OsSUV3 helicase. In this study a fresh batch of recombinant SUV3 protein from *Oryza sativa* (OsSUV3) was purified to homogeneity and characterized in detail using biochemical assays. ATPase assay in the presence of either DNA or RNA was performed and OsSUV3 exhibits ATP hydrolyzing properties. The OsSUV3 contains the DNA and RNA binding and DNA and RNA helicase activities. OsSUV3 exhibits bidirectional DNA unwinding activity and is unable to unwind the blunt-end DNA duplex substrate. The detailed OsSUV3 helicase kinetics was performed and the Km values are 0.51 and 0.95 nM for DNA helicase and RNA helicase, respectively. This study will be helpful in understanding the biochemical properties of OsSUV3 protein.

## Results

### Purification of OsSUV3 protein

The expression clone corresponding to *OsSUV3* was transformed into *E. coli* strain BL21 (DE3) pLysS and a fresh batch of his-tagged recombinant protein of 67 kDa (Additional file 1: Figure S1A) was purified as described earlier [13]. The purified protein was confirmed by western blotting with anti-His antibodies (Additional file 1: Figure S1B). This purified protein was used for all the characterization as described in the following sections.

### Characterization of ATPase activity of OsSUV3 protein

**Figure 1:**
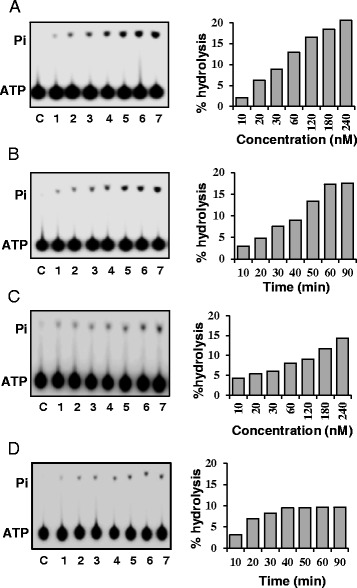
**ATPase activity of purified OsSUV3 in presence of DNA and RNA. (A)** ATPase activity of purified OsSUV3in the presence of ssDNA. Lanes 1–7, reactions with different concentration (10–240 nM) of enzyme. **(B)** Time dependence of ATPase activity of OsSUV3 (180 nM) in the presence of ssDNA. Lanes 1–7, reactions with enzyme at various time points. **(C)** ATPase activity of purified OsSUV3 in presence of RNA. Lanes 1–7, reactions with different concentration (10–240 nM) of enzyme. **(D)** Time dependence of ATPase activity of OsSUV3 (180 nM) in presence of RNA. Lanes 1–7, reactions with enzyme at various time points. Lane C in A-D shows reaction without enzyme. The quantitative data of the autoradiograms in A-D gels are shown as histograms on the right side of each autoradiogram.

The concentration-dependence of ssDNA-dependent ATPase activity was checked by using 10 to 240 nM of OsSUV3 protein and the percent release of radioactive inorganic phosphate (Pi) from [γ^32^P] ATP was measured. The results clearly show that OsSUV3 contains concentration-dependent ATPase activity in presence of ssDNA (Figure 1A, lanes 1–7). In order to study the time dependence of ATPase activity, the ATPase reaction using 180 nM of purified OsSUV3 at different time points was done. The percent release of radioactive Pi from [γ-^32^P] ATP showed linearity up to 60 minutes and the activity did not increase further on increasing the time of incubation (Figure 1B, lanes 1–7). The ATPase activity was also checked in the presence of RNA using the same concentrations of OsSUV3. It is interesting to note that OsSUV3 also exhibits RNA-dependent ATPase activity in concentration and time-dependent manner (Figure 1C, lanes 1–7 and Figure 1D, lanes 1–7, respectively).

### Characterization of DNA helicase activities of OsSUV3 protein

**Figure 2:**
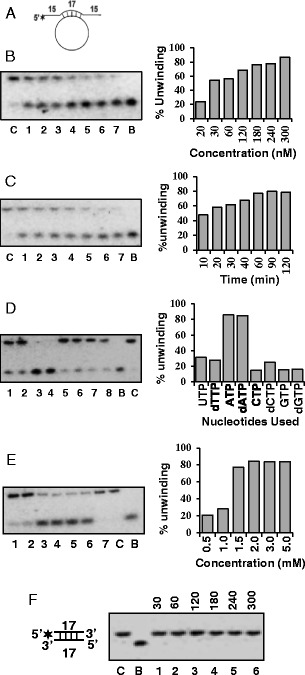
**DNA helicase activity analysis of OsSUV3 protein. (A)** The structure of the DNA helicase substrate used for assays in **B**-**E**. The substrate contains 15 nucleotide hanging tails at both the 5’ and 3’ ends. **(B)** Concentration-dependent helicase activity of purified OsSUV3, lanes 1–7 contain increasing concentration (20–300 nM) of OsSUV3. **(C)** Time dependent helicase activity of OsSUV3. Lanes 1–7, reactions with 250 nM of OsSUV3 at various time points. **(D)** Nucleotide requirement of helicase activity of OsSUV3. Lanes 1–8, helicase activity with 250 nM of OsSUV3 in the presence of various NTP or dNTPs written on the top of the autoradiogram. **(E)** Helicase activity with 250 nM of OsSUV3 using varying concentration of ATP. Lanes 1–6 are reactions with concentration of ATP written on the top of the autoradiogram and lane 7 is reaction without ATP. The quantitative enzyme activity data from the autoradiogram in **B**-**E** are shown as histograms on the right side. In all the **B**-**E** panels (left side), C lane is reaction without enzyme and B lane is heat denatured substrate. **(F)** Concentration-dependent DNA helicase activity of purified OsSUV3 protein with blunt-ended duplex DNA substrate (structure is shown in the left). Lanes 1-6 contain increasing concentration (30-300 nM) of OsSUV3 protein. Lane C is reaction without enzyme and B lane is heat denatured substrate.

The standard DNA strand-displacement assay was used in order to characterize the DNA unwinding activity of OsSUV3. The structure of the hanging tails substrate used for measuring the DNA helicase activity is shown Figure 2A. The results of the DNA unwinding activity using different concentration of purified OsSUV3protein (20 to 300 nM) is shown in Figure 2B. It is interesting to note that OsSUV3 exhibits the concentration-dependent helicase activity (Figure 2B, lanes 1–7). The time-dependent DNA helicase activity analysis was also done using 250 nM of OsSUV3 and the results indicate that OsSUV3 contains time-dependent helicase activity (Figure 2C, lanes 1–7).

Almost all the helicases have specific nucleotide requirement and the hydrolysis of nucleotide is tightly coupled to unwinding activity. Therefore the helicase activity of OsSUV3 protein (300 nM) was measured with different nucleotide and deoxynucleotide triphosphates (NTPs and dNTPs). The activity was maximum in presence of ATP and dATP (Figure 2D, lanes 3 and 4, respectively). Although to some extent OsSUV3 also showed little unwinding activity in the presence of other dNTPs and NTPs such as UTP, dTTP, CTP, dCTP, GTP and dGTP (Figure 2D, lanes 1, 2, 5–8, respectively) but OsSUV3 did not show any unwinding activity in the absence of any NTP or dNTP (Figure 2D, lane C). The concentration requirement using ATP showed that the unwinding activity of OsSUV3 (300 nM) was maximal at 2.0 mM ATP concentration and it did not increase further on increasing the ATP concentration to 5.0 mM (Figure 2E, lane 4, 5 and 6, respectively). The specificity of OsSUV3 was further determined by checking its DNA unwinding activity with blunt-ended duplex DNA substrate. This substrate has same duplex length (17 base pair) and identical core sequence as the hanging tails substrate but had blunt ends so that as far as possible, any differences in efficiency of unwinding due to sequence differences could be eliminated. The results clearly indicate that OsSUV3 protein is unable to unwind the blunt-ended duplex substrate (Figure 2F, lanes 1–6).

### Determination of direction of unwinding of OsSUV3 protein

**Figure 3:**
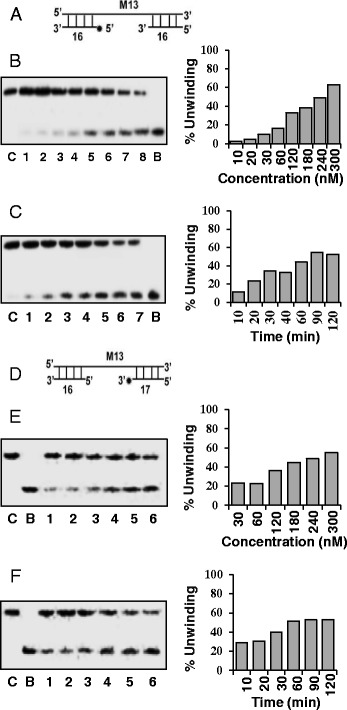
**Direction of DNA unwinding by OsSUV3 protein. (A)** The structure of the linear substrate for the measurement of 3’ to 5’ direction activity is shown. An asterisk (*) denotes the ^32^P-labeled end. The 3’ to 5’ direction specific DNA helicase activity of OsSUV3 protein using this substrate is shown in panels **B** and **C**. **(B)** Lanes 1–8 are reactions with increasing concentration (10–300 nM) of OsSUV3 protein. **(C)** Time-dependence of the helicase reaction with substrate shown. Lanes 1–7 are reactions with 250 nM of OsSUV3 at various time points. **(D)** The structures of the linear substrate for the 5’ to 3’ direction is shown. An asterisk (*) denotes the ^32^P-labeled end. The 5’ to 3’ direction specific DNA helicase activity of OsSUV3 protein using this substrate is shown in panels **E** and **F**. **(E)** Lanes 1–7 are reactions with increasing concentration (30–300 nM) of OsSUV3 protein. **(F)** Time-dependence of the helicase reaction with substrate shown. Lanes 1–6 are reactions with 250 nM of OsSUV3 at various time points written on top of the autoradiogram. The quantitative enzyme activity data from the autoradiogram in **B**-**C** and **E**-**F** are shown as histograms on the right side. In panels **B**, **C**, **E** and **F**, lane C is the reaction without enzyme and lane B is the heat-denatured substrate.

In a duplex DNA most of the helicases preferentially unwind in a polar fashion by moving unidirectionally on the bound strand. The strand to which the enzyme binds and moves defines the direction of unwinding by a helicase. The unwinding activity of purified OsSUV3 was tested by using the direction-specific substrates, one specific for the 3’ to 5’ (Figure 3A) and the other for the 5’ to 3’ direction (Figure 3D). Using both the direction-specific substrates the DNA unwinding activity of different concentrations (10–300 nM) of OsSUV3 was determined. The release of radiolabelled DNA fragment from the substrate of Figure 3A by OsSUV3 enzyme will indicate the movement in 3’ to 5’ direction. The results show that OsSUV3 is able to show the activity with the 3’ to 5’ direction-specific substrate in a concentration and time-dependent manner (Figure 3B and C, lanes 1–8 and lanes 1–7, respectively). By using 5’ to 3’ direction-specific duplex substrate (Figure 3D) the OsSUV3 also shows 5’ to 3” direction-specific activity in a concentration and time-dependent manner (Figure 3E, lanes 1–6 and Figure 3F, lanes 1–6, respectively). The results indicate that OsSUV3 protein contains bidirectional DNA unwinding activity.

### Determination of RNA helicase activity of OsSUV3 protein

**Figure 4:**
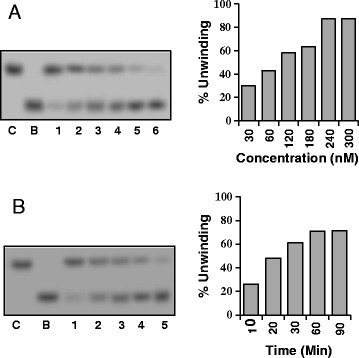
**RNA helicase activity of OsSUV3 protein. (A)** Concentration dependence of RNA helicase activity. Lanes 1–6 show the activity with different concentrations (30–300 nM) of purified OsSUV3. **(B)** Time dependence of RNA helicase activity. Lanes 1–6 show the activity at different time points with 200 nM of purified OsSUV3. In **A** and **B**, lane C is the reaction without enzyme and lane B is the heat-denatured substrate.

Using different concentrations of OsSUV3 protein and the partially duplex RNA-RNA substrate, the RNA helicase activity was checked. The results indicate that OsSUV3 protein (30–300 nM) contains concentration-dependent RNA-unwinding activity (Figure 4A, lanes 1–6). The unwinding activity was up to ~90%. The time-dependent RNA unwinding assay was also performed and the results show that OsSUV3 (200 nM) unwinds RNA duplex substrate in time-dependent manner with maximum activity achieved in one hour (Figure 4B, lanes 1–6) and the unwinding activity did not increase further.

### DNA and RNA binding activities of OsSUV3 protein

**Figure 5:**
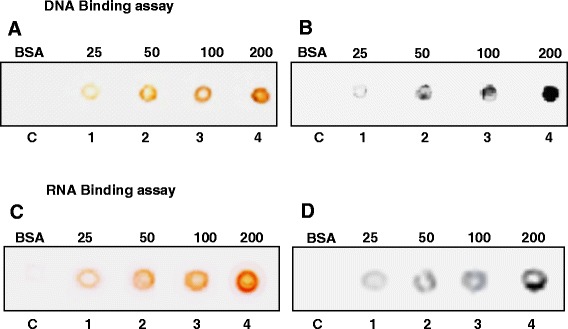
**DNA and RNA binding activity of OsSUV3.** DNA binding activity of OsSUV3. **(A)** Western blot probed with anti-his antibody. Different amounts (25–200 ng) of purified OsSUV3 (lanes 1–4) were spotted on the charged PVDF membrane and the blot was probed with anti-his antibody and developed using the standard procedure. **(B)** DNA binding activity. This experiment was done as written in the methods section. RNA binding activity of OsSUV3. **(C)** Western blot probed with anti-his antibody. Different amounts (25–200 ng) of purified OsSUV3 (lanes 1–4) were spotted on the charged PVDF membrane and the blot was probed with anti-his antibody and developed using the standard procedure. **(D)** RNA binding activity. This experiment was done as written in the methods section.

DNA-binding activity of OsSUV3 was checked using labeled DNA oligodeoxynucleotide. The results indicate that OsSUV3protein exhibits concentration-dependent DNA-binding activity (Figure 5B, lanes 1–4). BSA was used as a negative control, which showed no binding to the DNA (Figure 5B, lane C). The concentration-dependent loading of protein was confirmed by using an identical blot of OsSUV3, which was probed with anti-His antibody (Figure 5A, lanes 1–4). Similarly the RNA-binding activity of OsSUV3 was checked using labeled RNA oligodeoxynucleotide. The results indicate that OsSUV3also shows concentration-dependent RNA-binding activity (Figure 5D, lanes 1–4). BSA was used as a negative control, which showed no binding to the RNA (Figure 5D, lane C). The concentration-dependent loading of protein was confirmed by using an identical blot of OsSUV3, which was probed with anti-his antibody (Figure 5C, lanes 1–4).

### Determination of *K*_*m*_ and *V*_*max*_ for the helicase activity of OsSUV3 protein

**Figure 6:**
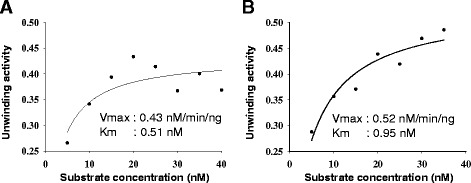
**Kinetics of DNA and RNA helicase activity.** Km and Vmax of DNA helicase **(A)**, and RNA helicase **(B)** activities of OsSUV3. The helicase reactions were carried out as reported in methods section and Km and Vmax were calculated from the plot.

Helicase assay reactions with OsSUV3 protein were performed using different concentrations (5–40 nM) of the DNA or RNA substrate in a standard reaction buffer. The quantification of amount of dsDNA and unwound ssDNA and dsRNA and unwound RNA was done as described in experimental procedures section. The rate of substrate unwinding was initially linear and later saturated with increasing substrate concentrations that gave best-fit to the Michaelis-Menten equation and a conventional hyperbolic dependence of the rate of reaction on substrate concentration was obtained. The K_m_ and V_max_ of DNA and RNA helicase activity for OsSUV3 was measured by using Sigma plot software (http://www.sigmaplot.com/). The nonlinear regression analysis of this data yielded a *Km* value of 0.51 nM for OsSUV3 DNA helicase (Figure 6A). The *Vmax* value for DNA helicase is 0.43 nM/min/ng for OsSUV3 (Figure 6A). The nonlinear regression analysis of the data yielded a *Km* value of 0.95 nM for OsSUV3 RNA helicase (Figure 6B). The *Vmax* value for RNA helicase is 0.52 nM/min/ng for OsSUV3 (Figure 6B).

## Discussion

In a recent study we have reported that OsSUV3 protein contains the highest sequence homology to SUV3 protein from *Arabidopsis thaliana*, as compared to its yeast and human counterparts [13]. AtSUV3 has been reported to be present in the mitochondria [12] therefore most likelyOsSUV3 is also present in mitochondria. In the present study we report the detailed biochemical characterization of OsSUV3 helicase. The results show that OsSUV3 exhibits DNA and RNA-dependent ATPase, DNA and RNA-binding and DNA and RNA unwinding activities. The results further show that OsSUV3 is a bipolar helicase capable of unwinding the DNA duplex in both the directions. Recently, the bipolar DNA helicase activity was also reported for pea p68 (Psp68) protein [14]. The plant pea DNA helicase 47 (PDH47) was also reported as bipolar DNA helicase [15]. Previously it has been reported that the some other non-plant helicases such as PfDH60, PfH45 and Dbp5/DDX19 homologue from human malaria parasite *Plasmodium falciparum* exhibit bipolar DNA helicase activity [16-19]. Some of the bacterial DNA helicases (PcrA and HerA) have also been reported as bipolar DNA helicases [20,21]. In some of the previous studies it was suggested that hSUV3 moves in 5’ to 3’ direction [9] but some other results show that hSUV3 moves along the substrate in 3’ to 5’ direction [10].

Further characterization reveals that OsSUV3 shows maximum DNA helicase activity only with ATP and dATP as compared to other NTPs/dNTPs. This characteristic was similar to previously reported pea DNA helicase 45 (PDH45) [22] and human DNA helicase II [23]. The AtSUV3 was reported to be localized in *A. thaliana* mitochondria. The characterization of the N-terminal domain of AtSUV3 containing the characteristic DExH-box helicase motifs revealed that it exhibited a low RNA stimulated ATPase activity in vitro [12]. Similar to OsSUV3 the hSUV3p is also reported to be associated with both the DNA and RNA helicase activities [6,9]. Our results indicate that OsSUV3 has no DNA unwinding activity with blunt end substrate. Previous studies have also reported that human and yeast SUV3 require a single-stranded fragment to unwind and have no detectable activity towards blunt-ended substrates [24]. It has been reported previously that hSUV3 interacts with the RPA (replication protein A) and FEN1 (flap endonuclease 1) which are RecQ helicase associated proteins. These observations suggest that even though SUV3 is considered a mitochondrial helicase but the physical and functional interactions between hSUV3 and RPA and FEN1 support the hypothesis that hSUV3 most likely plays an important role in nuclear DNA metabolism as well [10]. In an interesting recent study it has been reported that hSUV3, polynucleotide phosphorylase and mitochondrial polyadenylation polymerase form a transient complex to modulate mitochondrial mRNA polyadenylated tail lengths in response to energetic changes [25].

## Conclusions

The biochemical studies reported in this manuscript are the necessary first step to obtain new insights into enzyme function and regulation. Overall, this study is the first direct evidence to show the bipolar DNA helicase activity of OsSUV3 protein. This protein exhibits both the ATP-dependent DNA and RNA helicase activities and ssDNA/RNA-dependent ATPase activities which provide energy for its unwinding function. Since maintenance of mitochondrial DNA requires activity of RNA and DNA helicases, therefore the DNA and RNA helicase activity of OsSUV3 may be useful for mitochondrial RNA splicing, translation and genome maintenance. Through its RNA helicase property it may act as RNA chaperone to destabilize the inhibitory secondary structures especially during stress conditions in plants where secondary structures in RNA are common. Its bipolar DNA helicase activity could also be useful in normal functioning of the protein during the stress conditions. Overall these results suggest that OsSUV3 is most likely a multifunctional protein involved in diverse processes including nucleic acid metabolism and might be playing important roles in numerous physiological processes in plant.

## Methods

### Purification and characterization of OsSUV3

The cloning, expression and purification of OsSUV3 was done using the method described earlier [13]. The GenBank accession number of *OsSUV3* gene is GQ982584 (http://www.ncbi.nlm.nih.gov/nuccore/GQ982584) and the accession number of OsSUV3 protein sequence is ACX50964 (http://www.ncbi.nlm.nih.gov/protein/260800457). The purified protein was confirmed by SDS–PAGE analysis. This purified preparation was used for all of the enzyme assays.

### ATPase assay

The ATPase reaction was performed in the buffer (20 mM Tris–HCl, pH 8.0, 8 mM DTT, 1.0 mM MgCl_2_, 20 mM KCl and 16 μg/ml BSA) for 1 hour at 37°C in the presence of purified OsSUV3 and 10 ng of M13 mp19 ssDNA and a mixture of [γ-^32^P] ATP (~17 nM) and 1 mM cold ATP. The products were separated by thin layer chromatography (TLC) (33–35) and the plate was scanned on phosphoimager. The quantitation was done using IMAGE j/ geldoc software (http://rsbweb.nih.gov/ij/). For the concentration curve analysis different concentrations of OsSUV3 (from 10 to 240 nM) protein was used. The time course analysis was performed with a fixed concentration (180 nM) of OsSUV3 and time duration ranging from 10 to 90 minutes. The quantitation was done using IMAGE j/ geldoc software (http://rsbweb.nih.gov/ij/) and percentage of ATP hydrolysis was plotted as the bar diagram.

### Preparation of DNA helicase substrate and helicase assay

By using the standard strand displacement assay the helicase activity of OsSUV3 was determined using the partially duplex hanging tails substrate which consisted of a ^32^P-labelled 47-mer DNA oligodeoxynucleotide annealed to M13mp19 phage ssDNA. This oligodeoxynucleotide contains 15 base-pairs of non-complementary region at both 5’ and 3’ ends. Using T4 polynucleotide kinase (PNK) (5U) (New England Biolabs) in the standard PNK buffer (New England Biolabs) and 1.85 MBq of [γ-^32^P] ATP (specific activity 222 TBq/mmol)10 ng of the oligodeoxynucleotide was labeled at 5′-end at 37°C for one hour. Using 0.5 μg of single-stranded circular M13mp19 (+) phage DNA and standard annealing buffer (20 mM Tris–HCl, pH 7.5, 10 mM MgCl_2_, 100 mM NaCl, 1 mM DTT) the labeled oligodeoxynucleotide was annealed by heating at 95°C for 1 min, transferring immediately to 65°C for 2 min and then cooling slowly to room temperature. Using gel filtration through a Sepharose-4B column (Pharmacia, Sweden) the non-hybridized oligodeoxynucleotide was removed. The reaction mixture (10 μl) containing the ^32^P-labeled helicase substrate (1000 cpm/10 μl) in appropriate buffer (20 mM Tris–HCl, pH 8.0, 8 mM DTT, 1.0 mM MgCl_2_, 20 mM KCl and 16 μg/ml BSA), and the purified protein OsSUV3 was incubated at 37°C for 60 min. The substrate and products were separated by electrophoresis on a non denaturing 12% or 15% (for the blunt end substrate) PAGE, dried, and the gel was scanned on phosphoimager and both the substrate and unwound DNA bands were quantified. The quantitation was done using IMAGE j/ geldoc software (http://rsbweb.nih.gov/ij/) and the percent unwinding was plotted as the bar diagram.

### Preparation of RNA helicase substrate and unwinding assay

The RNA helicase substrate (RNA duplex) was prepared using the method described earlier [26]. The RNA helicase substrate was prepared by using the RNA oligonucleotides synthesized from Primm srl (Milan, Italy): 13 mer 5′-AUAGCCUCAACCG-3′ and 39 mer 5′-GGGAGAAAUCACUCGGUUGAGGCUAUCCGUAAAGCACGC-3′. Using five units of bacteriophage T4 polynucleotide kinase (NEB, England) about 10 ng of the 13-mer oligonucleotide was labeled at the 5′-end. This labeled oligonucleotide was then annealed with the 39-mer oligonucleotide using the standard procedure. The duplex RNA substrate was purified using the method described in the previous section. The RNA helicase assay (concentration and time course analysis) was done using the method described [26].

### Preparation of blunt-ended DNA helicase substrate

The sequence of 17 mer oligodeoxynucleotide used for making the blunt-ended duplex substrate is as follows 5’-GTTTTCCCAGTCACGAC-3’. This was labeled at 5’ end using the method described above and was annealed to its complementary oligodeoxynucleotide with the sequence 5’-GTCGTGACTGGGAAAAC-3’. The substrate was purified and used for the assay using the method described above.

### Preparation of direction specific substrates

The substrate consisting of long linear M13mp19 ssDNA with short duplex ends for 3’ to 5’ unwinding was prepared by first 5’-end labeling of 32-mer oligodeoxynucleotide and then annealing with M13mp19 ssDNA as described above. The annealed substrate was digested with SmaI and purified by gel filtration through 1 ml of Sepharose-4B.For preparing a 5’ to 3’ direction-specific substrate, the oligodeoxynucleotide 32-mer (5’-TTCGAGCTCGGTACCCGGGGATCCTCTAGAGT-3’) was first annealed to M13mp19 ssDNA using annealing buffer (20 mM Tris–HCl, pH 7.5, 10 mM MgCl_2_, 100 mM NaCl, 1 mM DTT) followed by labeling at 3’-OH end using standard buffer,5 units of DNA polymerase I (large fragment) and 50 μCurie [α-^32^P] dCTP at 23°C for 20 min. After increasing the dCTP to 50 mM using unlabelled dCTP the incubation was further continued for an additional 20 min at 23°C. The resulting duplex substrate was also digested with SmaI and purified by gel filtration through 1 ml Sepharose-4B.

### In vitro DNA/RNA binding assay

The DNA-binding assay was performed by using the end-labeled DNA oligodeoxynucleotide of 32 bases with the sequence 5’-TTCGAGCTCGGTACCCGGGGATCCTCTAGAGT-3’. BSA (1 μg) and different amounts of OsSUV3 were dot-blotted on pre-charged PVDF membrane and the membrane was incubated in blocking buffer which contained 25 mM NaCl, 10 mM MgCl_2_, 10 mM HEPES, 0.1 mM EDTA, 1 mM DTT and 3% BSA. The membrane was incubated for 2 hour in binding buffer containing 10 pmol of ^32^P-labeled DNA oligodeoxynucleotide after blocking. After binding, the membrane was washed thrice with binding buffer and exposed for autoradiography. Increasing amounts of OsSUV3 were dot-blotted on another precharged PVDF membrane to check for loading of proteins. This membrane was blocked with blocking buffer (1% BSA in TBS) for 1 hour at room temperature and probed for a further 1 hour with alkaline phosphatase conjugated anti-his antibody (Sigma Chemical Co) (St. Louis, MO, USA) in same buffer. The blot was washed and developed using standard protocol. The RNA binding activity was assayed using the similar method but the labeled RNA oligonucleotide with the sequence 13 mer 5′-AUAGCCUCAACC-G-3′ used for making the RNA substrate was used for the assay.

### Determination of Km and Vmax

Helicase assay reactions for OsSUV3 were performed using the substrate of different concentrations (5–40 nM) in a standard reaction buffer (20 mM Tris–HCl, pH 8.0, 8 mM DTT, 1.0 mM MgCl_2_, 20 mM KCl and 16 μg/ml BSA). Using ImageJ software (http://rsbweb.nih.gov/ij/) the amount of dsDNA and unwound ssDNA was quantified from the autoradiogram and used for the Km and Vmax calculations.

### Availability of supporting data

Name of the open access repository: NCBI (National Center for Biotechnology Information)

Link to the dataset for *OsSUV3* gene: http://www.ncbi.nlm.nih.gov/nuccore/GQ982584

Link to the dataset for OsSUV3 protein: http://www.ncbi.nlm.nih.gov/protein/260800457

## Additional file

Additional file 1: Figure S1. Protein purification and western blot of OsSUV3 protein. (A) Coomassie blue stained gel of purified OsSUV3. Lane M contains the protein molecular weight marker and lane 1 contains 0.2 μg of the purified OsSUV3. (B) Western blot of purified OsSUV3. Lane M contains the protein molecular weight marker and lane 1 contains 0.2 μg of the purified OsSUV3.
