# Supplementary material for: Rice SUV3 is a bidirectional helicase that binds both DNA and RNA
Source: BMC Plant Biol. 2014 Oct 14;14:283. doi: 10.1186/s12870-014-0283-6 (PMC4207899; doi:10.1186/s12870-014-0283-6)
Supplement: Additional file 1: Figure S1. — Protein purification and western blot of OsSUV3 protein. (A) Coomassie blue stained gel of purified OsSUV3. Lane M contains the protein molecular weight marker and lane 1 contains 0.2 μg of the purified OsSUV3. (B) Western blot of purified OsSUV3. Lane M contains the protein molecular weight marker and lane 1 contains 0.2 μg of the purified OsSUV3. [file 12870_2014_283_MOESM1_ESM.docx]

**Supplementary Information**

**
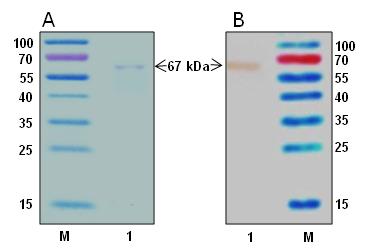
**

**Supplementary Figure S1. Protein purification and western blot of OsSUV3 protein.** (A) Coomassie blue stained gel of purified OsSUV3. Lane M contains the protein molecular weight marker and lane 1 contains 0.2 µg of the purified OsSUV3. (B) Western blot of purified OsSUV3. Lane M contains the protein molecular weight marker and lane 1 contains 0.2 µg of the purified OsSUV3.
